# A Comparative Evaluation of Advanced Platelet-Rich Fibrin and Titanium Platelet-Rich Fibrin in Maxillary Sinus Augmentation Using the Crestal Approach

**DOI:** 10.7759/cureus.90917

**Published:** 2025-08-24

**Authors:** Love K Bhatia, Kratika Rastogi, Shitij Srivastava, Natalia Trehan, Debajyoti Sarkar, Rashika Singh

**Affiliations:** 1 Department of Prosthodontics, Sardar Patel Post Graduate Institute of Dental and Medical Sciences, Lucknow, IND; 2 Department of Biomedical Sciences and Community Health, Division of Diagnostic Sciences, Medical University of South Carolina, Charleston, USA

**Keywords:** bone regeneration, dental implants, platelet-rich fibrin, prosthodontics, sinus floor augmentation

## Abstract

Aim and objectives

The present clinical investigation aimed to evaluate and compare the influence of advanced platelet-rich fibrin (A-PRF) and titanium platelet-rich fibrin (T-PRF) on implant stability and peri-implant bone density, following maxillary sinus augmentation through the crestal approach utilizing a bovine-derived xenograft. Both biomaterials are autologously derived and are increasingly used in implantology due to their regenerative potential. However, comparative data regarding their clinical performance in sinus lift procedures remain limited.

Materials and methods

This prospective, randomized in vivo clinical trial included 21 systemically healthy patients requiring bilateral crestal sinus augmentation, resulting in the placement of 42 dental implants. Each patient received two implants, one on each side of the maxilla, allocated using the Sequentially Numbered, Opaque, Sealed Envelope (SNOSE) method to ensure randomization and eliminate selection bias. Group A sites were treated with a combination of T-PRF and bovine xenograft (Bio-Oss; Geistlich Pharma AG, Wolhusen, Switzerland), while Group B received A-PRF, combined with the same xenograft material. Both types of PRF were prepared from the patients’ autologous blood, using their respective centrifugation protocols.

Patients were monitored for three months after sinus augmentation and implant placement. Implant stability was evaluated using resonance frequency analysis (RFA), expressed as the Implant Stability Quotient (ISQ). Bone density at the grafted sites was assessed using Hounsfield Units (HU) on cone-beam computed tomography (CBCT). All data were statistically analyzed using IBM SPSS Statistics for Windows, Version 21 (Released 2012; IBM Corp., Armonk, NY, USA), and intergroup comparisons were made using paired or independent t-tests. For all analyses, results with p-values less than 0.05 were regarded as statistically significant.

Results

The findings demonstrated that both A-PRF and T-PRF were effective in enhancing implant stability and promoting bone regeneration. However, T-PRF (Group A) showed significantly higher ISQ values and bone density measurements compared to A-PRF (Group B) at the three-month evaluation point. The improved clinical outcomes observed in the T-PRF group were consistent across all age groups, suggesting a more robust biological response. The denser fibrin matrix and longer-lasting release of growth factors associated with T-PRF may have contributed to its superior performance in terms of osseointegration and bone maturation.

Conclusions

The results of this study support the use of T-PRF as a more effective adjunct in sinus augmentation procedures via the crestal approach. When used in combination with a bovine xenograft, T-PRF leads to improved implant stability and greater bone density, compared to A-PRF. These findings suggest that T-PRF can enhance clinical outcomes and may serve as a preferred biomaterial in procedures aimed at achieving predictable and accelerated bone regeneration in the posterior maxilla. Further research, with larger cohorts and long-term follow-up, is recommended to validate these results and optimize treatment protocols.

## Introduction

Background and rationale

The introduction of platelet-rich plasma (PRP) in the late 20th century marked a significant advancement in regenerative dentistry, enhancing healing and osseointegration for dental implants. PRP, produced by centrifuging blood with anticoagulants, provides a concentrated supply of growth factors to surgical sites, accelerating tissue repair. In 2006, platelet-rich fibrin (PRF) emerged as a more biocompatible and effective alternative, requiring no anticoagulants or external activators. PRF develops into a fibrin matrix that entraps platelets, leukocytes, and various growth factors, enabling their sustained release over 7-14 days, thereby enhancing tissue regeneration and wound healing [1].

Advancements in platelet concentrate technology have introduced refined PRF variants, including advanced PRF (A-PRF) and titanium PRF (T-PRF). A-PRF, prepared via low-speed centrifugation, preserves higher platelet and leukocyte content, enhancing regenerative potential. T-PRF, produced using titanium tubes, improves platelet activation and fibrin quality, resulting in greater mechanical stability and accelerated tissue regeneration [2].

Crestal sinus lift surgery is a critical technique in implant dentistry for patients with insufficient posterior maxillary bone height. This minimally invasive approach addresses challenges of sinus pneumatization and post-extraction bone resorption [3]. T-PRF is effective in enhancing wound healing, promoting periodontal regeneration, supporting guided bone regeneration, facilitating sinus augmentation, and improving soft tissue regeneration around implants [4].

Objectives

The primary objective of this study is to compare the effects of A-PRF and T-PRF on implant stability following crestal sinus lift procedures. The secondary objective is to evaluate peri-implant bone density using Hounsfield units (HU) to assess the quality of bone regeneration. These objectives aim to provide evidence-based guidance for clinicians seeking to optimize sinus augmentation outcomes using PRF formulations.

## Materials and methods

Ethical approval

This clinical study was conducted in full accordance with the ethical principles outlined in the Declaration of Helsinki (revised 2013) [5]. Ethical clearance was obtained from the Institutional Ethical Committee (PROSTHO/04/222334/IEC). The study was also prospectively registered with the Clinical Trials Registry - India (CTRI) under the registration number (CTRI/2025/02/080033). Written informed consent was obtained from all participants after explaining the nature, benefits, and potential risks of the procedure in a language they could understand.

Patient and public involvement

Participants were fully informed about the study objectives, procedures, potential benefits, and risks before enrollment, and written informed consent was obtained. Patients were encouraged to ask questions and provide feedback throughout the study, particularly regarding their comfort and postoperative experiences. 

Trial design

**Figure 1:**
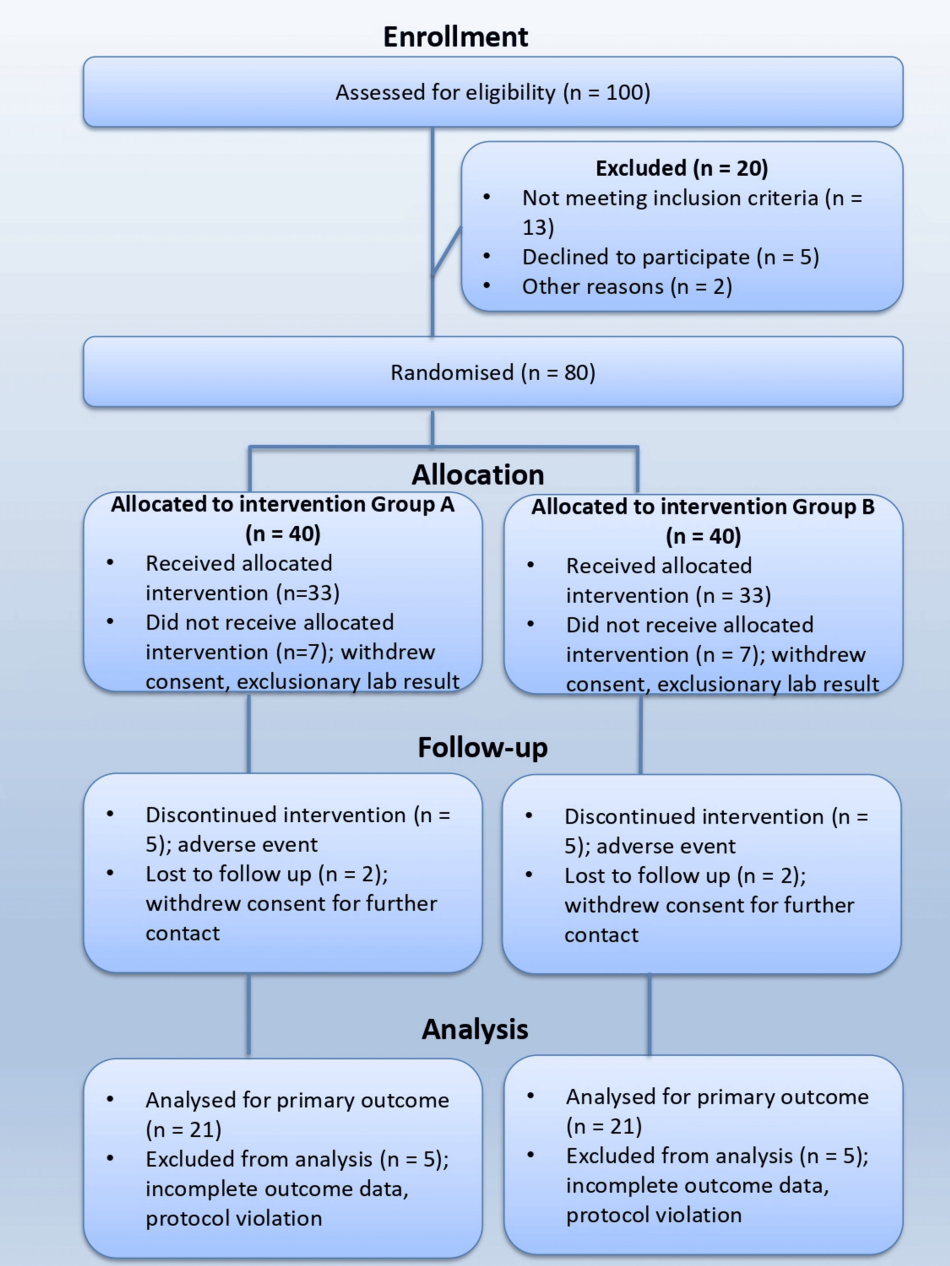
CONSORT 2025 flow diagram CONSORT 2025 flow diagram showing the progress of participants through the phases of the randomized trial, including enrollment, allocation, follow-up, and analysis. Reasons for exclusion, non-receipt of allocated intervention, discontinuation, and loss to follow-up are specified where applicable, in accordance with the CONSORT 2025 Statement [6]. CONSORT: Consolidated Standards of Reporting Trials

This study was a prospective, randomized, controlled, parallel-arm, single-blind, in vivo clinical trial with a split-mouth design, conducted following the CONSORT (Consolidated Standards of Reporting Trials) 2025 guidelines for reporting randomized trials (Figure 1) [6]. Each implant site was monitored for three months to assess the primary endpoint, with an overall study duration of six months, including analysis. The allocation ratio was 1:1, with each patient receiving both interventions (A-PRF and T-PRF) on the contralateral sides, allowing for intra-individual comparison. The trial was designed with a superiority framework to evaluate whether A-PRF or T-PRF provides improved implant stability and bone density following crestal sinus lift procedures. Participants were randomly assigned to two groups: Group A received the T-PRF intervention, and Group B received the A-PRF intervention.

Changes to trial protocol

No modifications were implemented in the study protocol once the trial had commenced. All primary and secondary outcomes, including Implant Stability Quotient (ISQ) and bone density (HU), were prespecified and analyzed as planned. Minor procedural adjustments, such as the timing of follow-up imaging, were made for logistical reasons without affecting outcome assessment or study integrity.

Trial setting

The trial was conducted in a tertiary care hospital setting, specifically within the Department of Prosthodontics, Sardar Patel Post Graduate Institute of Dental and Medical Sciences, Lucknow, India. All procedures and follow-up assessments were performed at a single institution, ensuring consistent surgical technique, postoperative care, and data collection across all participants.

Eligibility criteria

Participant Eligibility

Inclusion criteria include participants aged between 45 and 64 years with partially edentulous maxillary molar regions requiring implant placement and having residual alveolar bone height of 4 to 6 mm suitable for crestal sinus augmentation. All participants were systemically healthy, able to undergo oral surgery, and provided written informed consent. Patients were included irrespective of gender, socioeconomic status, caste, or religion.

Exclusion criteria included uncontrolled systemic diseases (e.g., diabetes mellitus), immunocompromised status, prior grafting at the implant site, active infection or pathology in the maxillary sinus or implant region, temporomandibular joint disorders, pregnancy or lactation, and poor oral hygiene, or inability to comply with postoperative instructions.

Personnel Eligibility

All surgical interventions were performed by a single, board-certified surgeon with extensive experience in implant placement and sinus augmentation procedures, thereby minimizing inter-operator variability and ensuring procedural consistency. Outcome assessments, including ISQ measurements via resonance frequency analysis (RFA) and peri-implant bone density evaluation using cone-beam computed tomography (CBCT), were conducted by an independent assessor, blinded to group allocation, to reduce detection bias and maintain objectivity in data collection.

Treatment protocol

Preoperative Assessment

A comprehensive preoperative evaluation was conducted for all participants, including a detailed medical and dental history to identify any contraindications for implant surgery. High-resolution imaging was performed using the CBCT system (Planmeca ProMax® 3D Mid, Helsinki, Finland), providing three-dimensional visualization of the maxillary sinus anatomy, bone volume, and density. This allowed accurate measurement of residual alveolar bone height and detection of sinus pathologies, such as mucosal thickening or cysts, which could complicate the procedure.

Implant Selection

Implant dimensions were individualized based on bone quality and width. Implants (MIS Implants Technologies Ltd., Misgav, Israel) with diameters of 4.0-5.0 mm and appropriate lengths were selected to ensure adequate primary stability while avoiding sinus membrane perforation, thereby optimizing conditions for osseointegration.

Interventions

Venous blood samples were collected from each participant under sterile conditions using 10 mL glass-coated plastic vacutainer tubes without anticoagulants for A-PRF. For T-PRF, blood was drawn into titanium-coated tubes to prevent clot adhesion to the tube walls and to promote better fibrin polymerization, platelet activation, and improved mechanical stability, along with prolonged release of growth factors.

**Figure 2:**
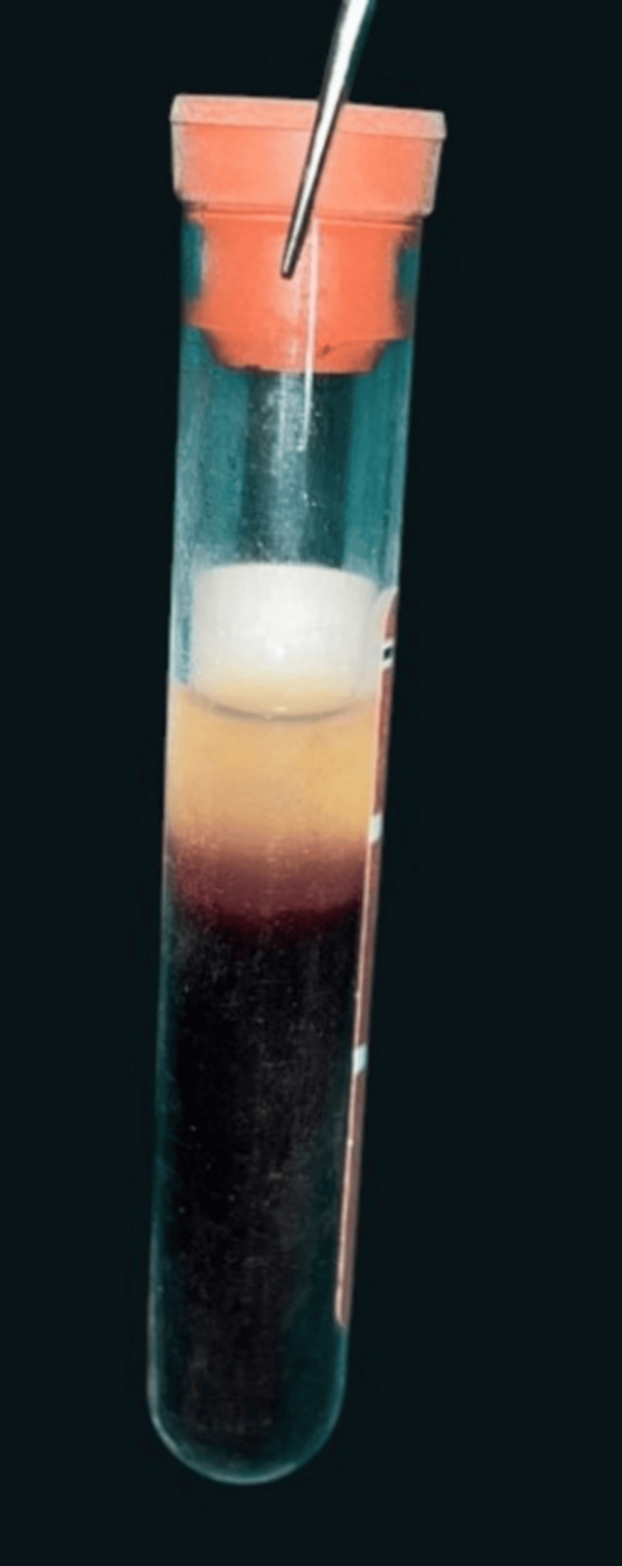
Preparation of A-PRF The A-PRF clot is gently removed from the centrifuge tube using forceps, prior to placement into the osteotomy site. The clot is ready for combination with a xenograft material for crestal sinus augmentation. A-PRF, Advanced Platelet-Rich Fibrin

A-PRF group: A-PRF was generated through low-speed centrifugation, performed at 1500 rpm for a duration of 14 minutes (Duo Quattro Centrifuge; DLAB Scientific Co., Ltd., Beijing, China) [7]. The resultant fibrin clot was mixed with a bovine-derived xenograft (Bio-Oss; Geistlich Pharma AG, Wolhusen, Switzerland) and placed at the osteotomy site during the crestal sinus lift (Figure 2).

**Figure 3:**
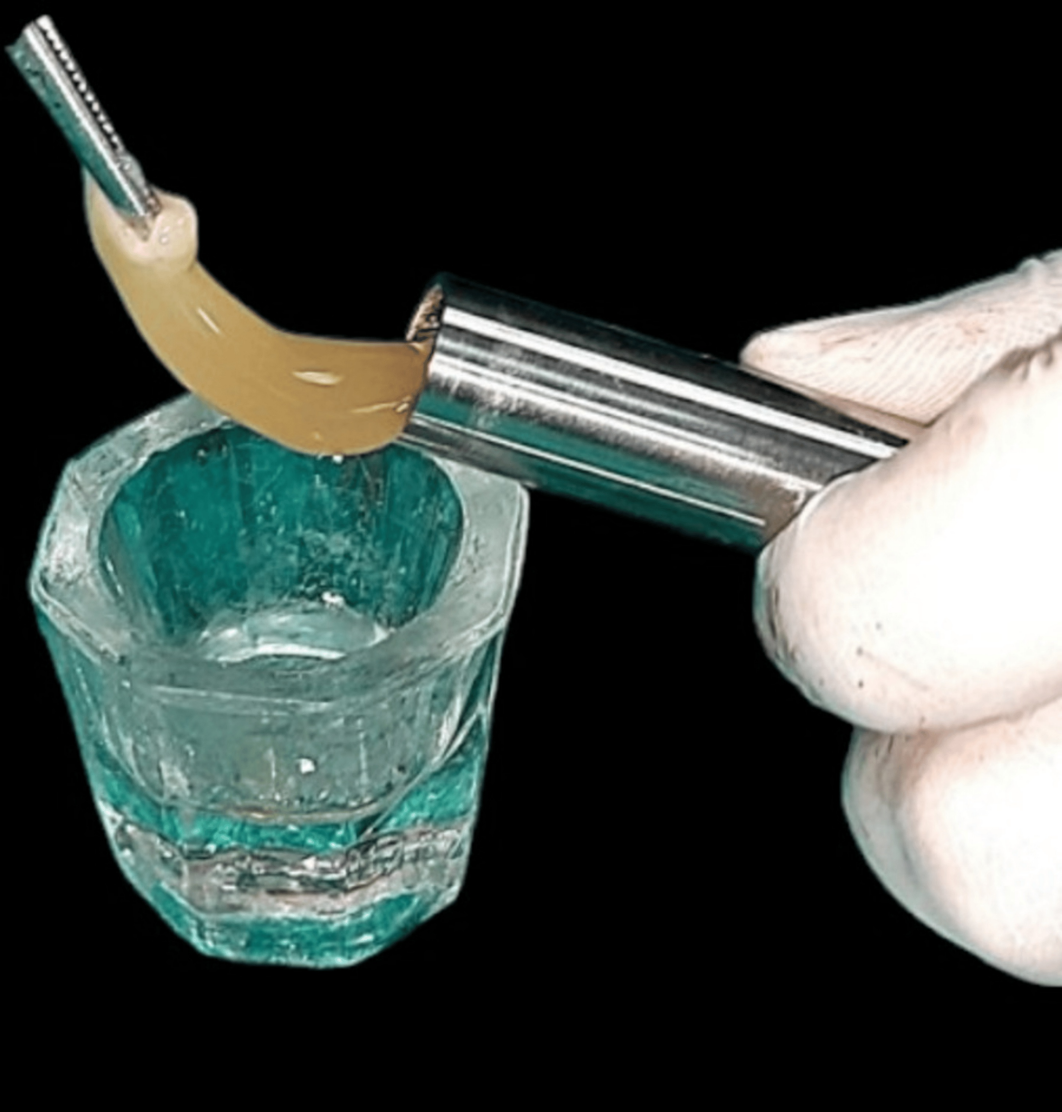
Preparation of T-PRF The T-PRF clot is gently removed from the titanium centrifuge tube using forceps, prior to placement into the osteotomy site. The clot is ready for combination with a xenograft material for crestal sinus augmentation. T-PRF, Titanium Platelet-Rich Fibrin

T-PRF group: T-PRF was prepared using titanium tubes subjected to centrifugation at 2800 rpm for 12 minutes (IntraSpin device; Intra-Lock International, Inc., Boca Raton, FL, USA) [4,8]. The T-PRF clot was similarly combined with a bovine-derived xenograft and used for sinus augmentation (Figure 3).

Both A-PRF and T-PRF were combined with the bovine-derived xenograft (Bio-Oss), a well-established osteoconductive scaffold, known to support new bone formation in sinus augmentation procedures [9]. This combination aims to leverage the biological advantages of PRF alongside the structural benefits of the bone graft material.

Replication Materials

Detailed procedural steps and PRF preparation protocols are described above and can be replicated following the specified centrifugation parameters, surgical instruments, and grafting protocol.

Surgical Procedure

Surgeries were performed under local anesthesia with Xylocaine (2% lidocaine with 1:100,000 epinephrine; AstraZeneca, Cambridge, UK), ensuring patient comfort and hemostasis. A crestal incision was made to access the alveolar ridge, and osteotomy sites were prepared using Densah burs (Versah LLC, Chicago, IL, USA) [10]. These specially designed burs facilitate atraumatic bone compaction and sinus membrane elevation by stopping 1-2 mm short of the sinus floor, thereby minimizing the risk of membrane perforation. 

**Figure 4:**
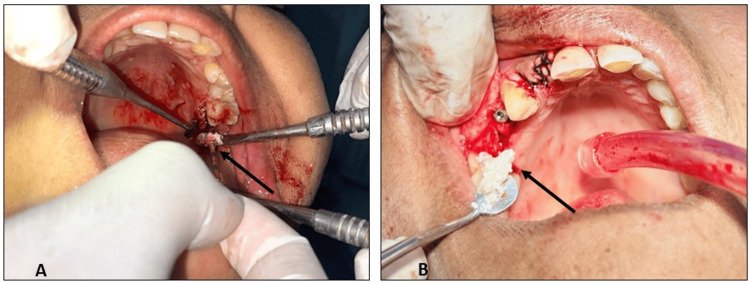
Compaction and placement of T-PRF and A-PRF with bone graft (A) Arrow indicates the placement of the T-PRF mixed with the bovine-derived xenograft into the surgical site, followed by the placement of implants. (B) Arrow indicates the placement of the A-PRF mixed with the bovine-derived xenograft into the surgical site, followed by the placement of implants. A-PRF, Advanced Platelet-Rich Fibrin; T-PRF, Titanium Platelet-Rich Fibrin

Following osteotomy preparation, A-PRF or T-PRF combined with bovine-derived xenograft was carefully compacted into the osteotomy site (Figure 4). The compaction process involved gently inserting and condensing the PRF-xenograft mixture against the sinus floor using bone-condensing instruments, creating a stable scaffold for new bone formation. Once the grafted site was adequately filled and compacted, dental implants of predetermined diameter and length were inserted into the prepared osteotomy, achieving primary stability. The combination of PRF compaction and precise implant placement promotes enhanced osseointegration and supports early bone regeneration within the augmented sinus region.

Postoperative Instructions

Postoperative care involved the prescription of antibiotics and the use of chlorhexidine mouth rinse to minimize infection risk. Patients were additionally advised to apply cold compresses for edema control, follow pain management protocols, and maintain oral hygiene. Sutures were removed on the 10th postoperative day, and patients were scheduled for regular follow-ups to monitor healing and evaluate implant stability.

Outcomes

Primary Outcome

ISQ is measured by RFA using the Osstell ISQ device (Osstell AB, Gothenburg, Sweden) [11-14]. The analysis metric is the mean ISQ at baseline and at three months post-implant placement.

Secondary Outcome

Peri-implant bone density was assessed in HU via CBCT imaging [15-17]. The analysis metric is the mean HU at baseline and three months post-surgery.

All outcome measures were prespecified, and results were aggregated as means ± standard deviation for each group at defined time points.

Harms

Harms included any intraoperative or postoperative complications such as sinus membrane perforation, infection, graft displacement, bleeding, or adverse reactions to PRF or graft materials. Complications were monitored systematically at each follow-up visit. Adverse events were recorded, categorized by severity, and reported according to standard clinical trial protocols.

Sample size

Determination of Sample Size

The sample size for this study was calculated using the formula suggested by Charan and Biswas (2013) [18]: $n = \frac{(r+1)}{r} \times \frac{(Z_{\alpha/2} + Z_{\beta})^2 \times SD^2}{d^2} = \frac{(1+1)}{1} \times \frac{(1.96 + 0.84)^2 \times (0.37)^2}{(0.36)^2} = 16.56 \approx 17$. Keeping a provision of data loss at 20%, the proposed minimum sample size is 21 patients in each group.

Interim Analyses and Stopping Guidelines

No interim analyses were planned, and there were no predefined stopping guidelines, as the trial was exploratory in nature with a short follow-up period.

Randomization

Sequence Generation

The random allocation sequence for assigning implant sites to either the A-PRF or T-PRF group was generated by an independent statistician not involved in patient recruitment or clinical procedures. A computer-generated sequence was used to achieve randomization with an allocation ratio of 1:1. To maintain equal distribution between groups and reduce the risk of selection bias, block randomization with variable block sizes of four and six was adopted. Stratification was deemed unnecessary since, in the split-mouth design, each participant functioned as their own control.

Allocation Concealment Mechanism

Allocation was implemented using the Sequentially Numbered, Opaque, Sealed Envelope (SNOSE) method [19]. Each envelope contained the assigned intervention for a specific implant site and was opened only at the time of surgery, immediately prior to graft placement, to maintain allocation concealment.

Implementation

The envelopes were prepared by a research coordinator independent of the surgical team. The enrolling clinician assigned the interventions according to the sequence provided in the sealed envelopes, without prior knowledge of allocation, ensuring unbiased treatment assignment.

Blinding

This was a single-blind trial with blinded outcome assessment. The assessor responsible for ISQ measurements and CBCT-based bone density evaluation was blinded to the allocation of participants. Blinding of the operating surgeon and participants was not feasible due to the physical differences in preparation and handling of A-PRF and T-PRF. To minimize bias, both materials were prepared and compacted using standardized protocols.

Method of checking ISQ and bone quality

Assessment of ISQ

**Figure 5:**
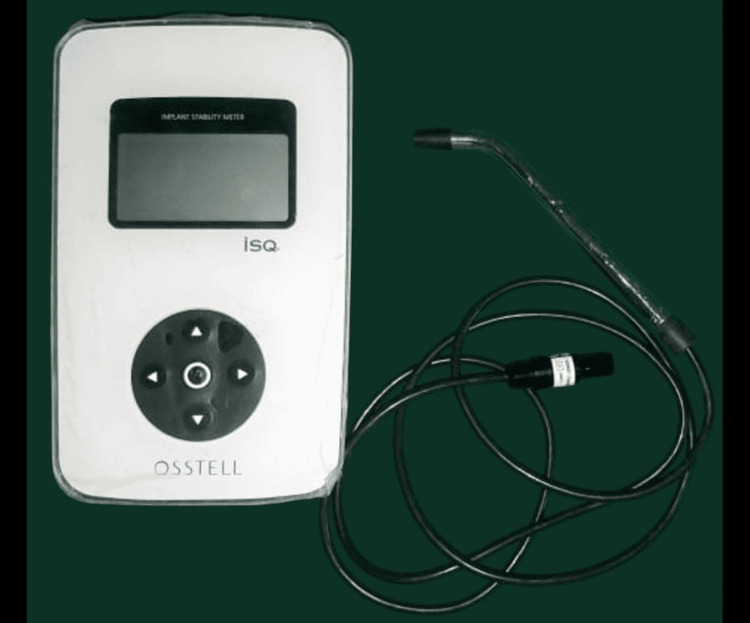
Osstell device It is used for the assessment of the Implant Stability Quotient (ISQ).

A non-invasive method for assessing implant stability involves the use of RFA, where vibrational waves are applied to a small transducer or peg attached to the implant (Figure 5). The frequency at which the system resonates provides insight into the level of stability within the surrounding bone. Typically, resonance frequency values fall within the range of 3500-8500 kHz, as initially described by Meredith et al. These values can be converted into a unitless scale ranging from 1 to 100, which helps clinicians interpret stability more easily [11-14].

Lower values on this scale (usually below 55) may indicate a higher likelihood of lateral movement and insufficient secondary stability, suggesting that the implant may not yet be ready for loading. An increase in resonance frequency measurements over time is often interpreted as a sign of progressing osseointegration. Monitoring these values, in conjunction with clinical and radiographic findings, allows clinicians to make informed decisions about the timing of prosthetic loading. If stability measurements remain low or fail to improve, the implant may be considered at risk.

Assessment of Bone Density Using CBCT: Standardization, Calibration, and Reliability Assessment

HU derived from CT provides a quantitative measure of bone density by assessing X-ray attenuation within each voxel. Medical CT is widely regarded as a dependable modality for HU-based evaluation of bone density; however, CBCT may yield inconsistent HU readings due to variations in equipment design, voxel dimensions, imaging field, and reconstruction algorithms. Therefore, careful standardization and validation are essential to improve reliability [20].

CBCT standardization in three planes: To ensure reproducibility, CBCT scans were standardized along three orthogonal planes - axial, coronal, and sagittal - using consistent patient positioning and fixed anatomical reference points. The occlusal plane was aligned parallel to the horizontal reference line to minimize rotational discrepancies. Cross-sectional slices of 0.2-0.3 mm voxel thickness were acquired to optimize spatial resolution while maintaining consistent imaging parameters across all subjects.

Scanner calibration: A phantom with known density materials (e.g., hydroxyapatite and water) was scanned prior to patient imaging to calibrate HU measurements. The linear attenuation coefficients of these reference materials allowed conversion of CBCT gray values to approximate HU values, mitigating scanner-specific biases [21]. Each imaging session was preceded by calibration scans to standardize and ensure consistency of measurements.

Measurement procedure and region of interest (ROI) selection: Bone density measurements were taken within standardized ROIs placed at the implant sites. Each ROI included only trabecular or cortical bone, as per the study protocol, avoiding artifacts from metallic restorations or air spaces. The mean HU value within the ROI was recorded from axial slices and cross-verified in sagittal and coronal reconstructions to ensure three-dimensional consistency.

Reliability assessment: To evaluate intra- and inter-observer reliability, two independent observers performed HU measurements on a subset of 10 randomly selected scans. Intra-class correlation coefficients (ICCs) were calculated, with values above 0.85 considered indicative of high reliability. Repeated measurements were taken at a two-week interval to assess measurement reproducibility.

Limitations and considerations: Despite these standardization steps, CBCT-derived HU values remain relative rather than absolute density measures. Factors such as beam hardening, scatter, and reconstruction algorithms may introduce variability. Therefore, interpretation of CBCT HU values requires caution and should ideally be complemented by clinical examination and histological assessment of bone characteristics.

**Figure 6:**
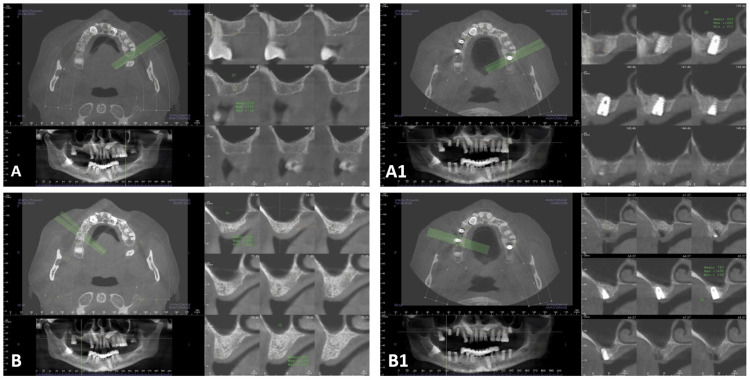
Pre and Post-op CBCT for T-PRF and A-PRF (A) CBCT baseline reading for T-PRF; (A1) CBCT 3-month follow-up reading for T-PRF. (B) CBCT baseline reading for A-PRF; (B1) CBCT 3-month follow-up reading for A-PRF. CBCT, Cone-Beam Computed Tomography; A-PRF, Advanced Platelet-Rich Fibrin; T-PRF, Titanium Platelet-Rich Fibrin

By implementing rigorous calibration, ROI standardization in three planes, and reliability testing, the present study aimed to optimize CBCT-based bone density evaluation [22], while acknowledging inherent limitations in absolute HU quantification (Figure 6).

Outcome assessment

To minimize assessment bias, the surgeon performing the procedures was aware of group allocation, whereas the outcome assessor was blinded to the type of PRF used at each implant site. Implant stability was measured using RFA with the Osstell ISQ device, which provides an ISQ value [11]. Bone density was evaluated quantitatively in HU using preoperative and postoperative CBCT scans [23].

Statistical methods

Primary and Secondary Outcomes

IBM SPSS Statistics for Windows, Version 21 (Released 2012; IBM Corp., Armonk, NY, USA), was employed for conducting the statistical analyses. Continuous variables were expressed as mean ± standard deviation (SD). The Shapiro-Wilk test was used to assess the normality of distribution for ISQ and bone density values. Since the data were normally distributed (p > 0.05), parametric tests were applied: paired t-test for within-group comparisons (baseline vs. three months), independent t-test for gender- and age-wise intergroup comparisons, and one-way analysis of variance (ANOVA) for intragroup age-wise analysis. A p-value < 0.05 was considered statistically significant.

Analysis Population

All randomized implant sites were included in the primary analysis according to the intention-to-treat principle. Each site was analyzed in the group to which it was originally allocated, regardless of deviations from the assigned intervention.

Handling of Missing Data

Missing outcome data were minimal; any missing ISQ or HU values due to patient dropout or imaging artifacts were addressed using last observation carried forward (LOCF) imputation. Sensitivity analyses confirmed that the imputation did not alter the overall findings.

## Results

The age of patients ranged from 45 to 64 years; the mean age was 55.38 ± 6.03 years. The maximum number of patients was in the 51-60 age group, i.e., eight (38.1%); six (28.6%) patients were aged above 60 years, and the remaining seven (33.3%) were aged ≤50 years (Table 1).

**Table 1:** Age-wise distribution of study population (n = 21) Distribution of participants by age group with corresponding frequencies and percentages. The mean age ± standard deviation (SD) and age range are also presented.

S. No.	Age group	Number (n)	Percentage
1	≤50 years	7	33.3
2	51-60 years	8	38.1
3	>60 years	6	28.6
Mean age ± SD (range)		55.38 ± 6.03 (45-64) years	

A slight dominance of females was observed in the study population (11, or 52.4%). The gender ratio (M:F) was 0.9 (Table 2).

**Table 2:** Gender wise distribution of study population (n = 21) Distribution of participants according to gender with corresponding numbers (n) and percentages.

S. No.	Gender	Number (n)	Percentage
1	Male	10	47.6
2	Female	11	52.4

The study, which used a split-mouth design, included 21 edentulous patients in each group (Table 3).

**Table 3:** Group wise distribution of patients Distribution of implants among study groups with the number and percentage of implants in each group. A-PRF, Advanced Platelet-Rich Fibrin; T-PRF, Titanium Platelet-Rich Fibrin

S. No.	Group	Number of implants	Percentage
1	Group A: T-PRF	21	50.0
2	Group B: A-PRF	21	50.0

Normality testing

The distribution of continuous variables, including ISQ and bone density values, was examined using the Shapiro-Wilk test. No significant deviation from normality was observed at baseline or at three-month follow-up in either group (p > 0.05). Accordingly, the data were considered normally distributed, and parametric statistical tests were applied for further analysis.

The mean ISQ for Group A, at baseline (Day 0), was 57.95 ± 3.22, which increased to 75.29 ± 2.43 after three months of follow-up. Paired t-test analysis revealed that this increase in ISQ values over time was statistically significant (p < 0.001), indicating a marked improvement in implant stability during the osseointegration period (Table 4).

**Table 4:** Comparison of ISQ for Group A at baseline and 3 months follow-up ISQ, Implant Stability Quotient

Time point	Mean ± SD
Day 0 (baseline)	57.95 ± 3.22
After 3 months	75.29 ± 2.43

The mean ISQ for Group B, at baseline (Day 0), was 55.52 ± 3.98, which increased to 70.94 ± 1.91 after three months of follow-up. Paired t-test analysis revealed that this increase in ISQ values over time was statistically significant (p < 0.001), indicating a marked improvement in implant stability during the osseointegration period (Table 5).

**Table 5:** Comparison of ISQ for Group B at baseline and 3 months follow-up ISQ, Implant Stability Quotient

Time point	Mean ± SD
Day 0 (baseline)	55.52 ± 3.98
After 3 months	70.94 ± 1.91

After three months of implantation, none of the patients in either group had an ISQ ≤ 55. The minimum ISQ in Group A was 72, while that in Group B was 70. The maximum ISQ in Groups A and B were 80 and 78, respectively. The mean ISQ of Group A was significantly higher than that of Group B (75.29 ± 2.43 vs. 70.94 ± 1.91; p < 0.001) (Table 6).

**Table 6:** Comparison of ISQ of two study groups after 3 months of implantation Comparison of implant stability values (ISQ scores) between Group A (T-PRF) and Group B (A-PRF). Values are presented as minimum, maximum, median, mean, and standard deviation (SD). Statistical analysis was performed using the paired t-test (t = 5.148; p < 0.001), indicating a statistically significant difference between the groups. ISQ, Implant Stability Quotient; A-PRF, Advanced Platelet-Rich Fibrin; T-PRF, Titanium Platelet-Rich Fibrin

S. No.		Group A	Group B	Total implants
1	n	21	21	42
2	Minimum	72.0	70.0	70.0
3	Maximum	80.0	78.0	80.0
4	Median	75.0	72.0	73.0
5	Mean	75.29	70.94	73.55
6	SD	2.43	1.91	2.79
	Paired t-test	t = 5.148; p < 0.001		

The mean bone density for Group A at baseline (Day 0) was 459.90 ± 23.11 HU, which increased to 685.95 ± 6.70 HU after three months of follow-up. Paired t-test analysis revealed that this increase in bone density values over time was statistically significant (p < 0.001), indicating a substantial improvement in peri-implant bone density during the healing period (Table 7).

**Table 7:** Comparison of bone density (Hounsefield Unit) for Group A at baseline and 3 months follow-up

Time point	Mean ± SD
Day 0 (baseline)	459.90 ± 23.11
After 3 months	685.95 ± 6.70

The mean bone density for Group B, at baseline (Day 0), was 458.10 ± 23.23 HU, which increased to 639.62 ± 9.89 HU after three months of follow-up. Paired t-test analysis revealed that this increase in bone density values over time was statistically significant (p < 0.001), indicating a considerable enhancement in peri-implant bone density during the osseointegration period (Table 8).

**Table 8:** Comparison of bone density (Hounsefield Unit) for Group B at baseline and 3 months follow-up

Time point	Mean ± SD
Day 0 (baseline)	458.10 ± 23.23
After 3 months	639.62 ± 9.89

After three months of implant placement, the bone density of Group A ranged from 671 to 698 HU, while that of Group B ranged from 630 to 668 HU. The median bone density of Groups A and B was 688 and 638, respectively. The difference in mean bone density of Group A (685.95 ± 6.70 HU) and that of Group B (639.62 ± 9.89 HU) was found to be statistically significant (Table 9).

**Table 9:** Comparison of bone density (Hounsfield unit) of two study groups after 3 months follow-up Comparison of bone density measurements (in Hounsfield Units) between Group A (T-PRF) and Group B (A-PRF). Data includes minimum, maximum, median, mean, and standard deviation (SD) values. Statistical analysis using a paired t-test (t = 17.768; p < 0.001) indicates a highly significant difference between the groups. A-PRF, Advanced Platelet-Rich Fibrin; T-PRF, Titanium Platelet-Rich Fibrin

S. No.		Group A	Group B	Total implants
1	n	21	21	42
2	Minimum	671.0	630.0	630.0
3	Maximum	698.0	668.0	698.0
4	Median	688.0	638.0	669.50
5	Mean	685.95	639.62	662.79
6	SD	6.70	9.89	24.89
	Paired t-test	t = 17.768; p < 0.001		

In Group A, the ISQ was higher in males (76.10 ± 2.23) compared to females (74.55 ± 2.46), but this difference was not statistically significant. Similarly, in Group B, males had a higher ISQ (72.10 ± 2.23) than females (71.55 ± 1.63), though this difference was also not statistically significant. Within-group comparisons of implant stability between males and females were not significant for either group. However, Group A showed significantly higher ISQ values compared to Group B for both genders: males (76.10 ± 2.23 vs. 72.10 ± 2.23) and females (74.55 ± 2.46 vs. 71.55 ± 1.63) (Table 10).

**Table 10:** Gender wise comparison of ISQ of two study groups Gender-wise comparison of implant stability values (ISQ scores) within Group A (T-PRF) and Group B (A-PRF). The data include the mean and standard deviation (SD) for male and female participants in each group. ISQ, Implant Stability Quotient; A-PRF, Advanced Platelet-Rich Fibrin; T-PRF, Titanium Platelet-Rich Fibrin

No.	Gender	Group A (Mean ± SD)	Group B (Mean ± SD)	Statistical significance	Test applied
1	Male (n = 10)	76.1 ± 2.23	72.1 ± 2.23	t = 4.004; p = 0.001	Paired t-test
2	Female (n = 11)	74.55 ± 2.46	71.55 ± 1.63	t = 3.365; p = 0.003	Paired t-test
3	Gender-wise difference within Group A	Male: 76.1 ± 2.23 vs. Female: 74.55 ± 2.46	-	t = 1.509; p = 0.148	Independent t-test
4	Gender-wise difference within Group B	-	Male: 72.1 ± 2.23 vs. Female: 71.55 ± 1.63	t = 0.654; p = 0.521	Independent t-test

In Group A, the bone density (HU) of males (687.70 ± 7.64) was slightly higher than that of females (684.36 ± 5.6), but this difference was not statistically significant. Similarly, in Group B, the difference in bone density between males (642.80 ± 11.30) and females (636.73 ± 7.83) was not significant. Within-group comparisons of bone density between males and females were not significant for either group. However, Group A had significantly higher bone density compared to Group B for both genders: males (687.70 ± 7.64 vs. 642.80 ± 11.30) and females (684.36 ± 5.6 vs. 636.73 ± 7.83) (Table 11).

**Table 11:** Gender wise comparison of bone density of two study groups Gender-wise comparison of bone density values (in Hounsfield Units) between Group A (T-PRF) and Group B (A-PRF). The table presents mean and standard deviation (SD) for male and female participants in each group. A-PRF, Advanced Platelet-Rich Fibrin; T-PRF, Titanium Platelet-Rich Fibrin

No.	Gender/Comparison	Group A (mean ± SD)	Group B (mean ± SD)	Statistical significance	Test applied
1	Male (n = 10)	687.7 ± 7.64	642.8 ± 11.3	t = 10.406; p < 0.001	Paired t-test
2	Female (n = 11)	684.36 ± 5.6	636.73 ± 7.83	t = 16.394; p < 0.001	Paired t-test
3	Gender-wise difference within Group A	Male: 687.7 ± 7.64 vs Female: 684.36 ± 5.6	-	t = 1.133; p = 0.273	Independent t-test
4	Gender-wise difference within Group B	-	Male: 642.8 ± 11.3 vs Female: 636.73 ± 7.83	t = 1.417; p = 0.176	Independent t-test

A statistically significant difference in ISQ was observed between Group A and Group B across all age groups. Group A demonstrated significantly higher ISQ than Group B in the age groups ≤50 years (76.14 ± 2.61 vs. 72.29 ± 2.75), 51-60 years (74.88 ± 2.70 vs. 71.38 ± 1.41), and >60 years (74.83 ± 1.94 vs. 71.83 ± 1.47). Younger patients (≤50 years) showed higher implant stability compared to older patients (51-60 years and >60 years) in both groups, although this difference was not statistically significant: Group A (76.14 ± 2.61 vs. 74.88 ± 2.70 vs. 74.83 ± 1.94) and Group B (72.29 ± 2.75 vs. 71.38 ± 1.41 vs. 71.83 ± 1.47) (Table 12).

**Table 12:** Age-wise comparison of ISQ of the two study groups Age-wise comparison of implant stability values (ISQ scores) between Group A (T-PRF) and Group B (A-PRF). The table presents the mean and standard deviation (SD) for each age group. Intergroup comparisons between Group A and Group B within each age group were performed using a paired t-test to account for the split-mouth design, while intragroup age-wise differences within each group were assessed using one-way ANOVA, which revealed no statistically significant variation within the groups (p > 0.05). ISQ, Implant Stability Quotient; A-PRF, Advanced Platelet-Rich Fibrin; T-PRF, Titanium Platelet-Rich Fibrin; ANOVA, Analysis of Variance

S. No.	Age group	Group A		Group B		Statistical significance
		n	Mean ± SD	n	Mean ± SD	
1	≤50 years	7	76.14 ± 2.61	7	72.29 ± 2.75	t = 2.691; p = 0.020
2	51-60 years	8	74.88 ± 2.7	8	71.38 ± 1.41	t = 3.255; p = 0.006
3	>60 years	6	74.83 ± 1.94	6	71.83 ± 1.47	t = 3.017; p = 0.013
Age-wise difference within group (ANOVA)		F = 0.628; p = 0.545		F = 0.398; p = 0.677		-

On comparing the within-group bone density (HU) of patients across different age groups (≤50 years, 51-60 years, and >60 years), no statistically significant differences were found. In Group A, bone densities were 688.29 ± 5.06, 683.38 ± 5.48, and 686.67 ± 9.40 (p = 0.369), and in Group B, they were 643.00 ± 13.63, 635.75 ± 3.45, and 640.83 ± 10.50 (p = 0.363). While younger patients (≤50 years) had higher bone density compared to older patients, this difference was not statistically significant. However, bone density in Group A was significantly higher than that of Group B in each age group: ≤50 years (688.29 ± 5.06 vs. 643.00 ± 13.63), 51-60 years (683.38 ± 5.48 vs. 635.75 ± 3.45), and >60 years (686.67 ± 9.40 vs. 640.83 ± 10.50) (Table 13).

**Table 13:** Age wise comparison of bone density of two study groups Age-wise comparison of bone density values (in Hounsfield Units) between Group A (T-PRF) and Group B (A-PRF). The table shows the mean and standard deviation (SD) for each age group. Intergroup comparisons between Group A and Group B within each age group were performed using a paired t-test, which revealed statistically significant differences across all age groups (p < 0.001). Intragroup age-wise comparisons within each group were assessed using one-way ANOVA, which showed no statistically significant differences (p > 0.05). A-PRF, Advanced Platelet-Rich Fibrin; T-PRF, Titanium Platelet-Rich Fibrin; ANOVA, Analysis of Variance

S. No.	Age group	Group A		Group B		Statistical significance
		n	Mean ± SD	n	Mean ± SD	
1	≤50 years	7	688.29 ± 5.06	7	643 ± 13.63	t = 8.244; p < 0.001
2	51-60 years	8	683.38 ± 5.48	8	635.75 ± 3.45	t = 20.807; p < 0.001
3	>60 years	6	686.67 ± 9.4	6	640.83 ± 10.5	t = 7.970; p < 0.001
Age-wise difference within group (ANOVA)		F = 1.055; p = 0.369		F = 1.074; p = 0.363		-

## Discussion

This split-mouth, randomized controlled trial evaluated the comparative efficacy of T-PRF and A-PRF in enhancing implant stability and peri-implant bone density in the posterior maxilla over three months. Our findings demonstrate that T-PRF significantly outperforms A-PRF in both implant stability and bone density, irrespective of age and gender subgroups.

Implant stability analysis

The ISQ values increased significantly in both groups. Group A (T-PRF) increased from 57.95 ± 3.22 at baseline to 75.29 ± 2.43 at three months, while Group B (A-PRF) increased from 55.52 ± 3.98 to 70.94 ± 1.91 (p < 0.001). Intergroup comparison favored T-PRF (p < 0.001), confirming the superior osseointegration observed in the study by Aldommari et al. [24].

A gender-wise analysis showed a slightly higher ISQ in males, but this difference was not statistically significant within groups. Intergroup comparisons for both genders demonstrated higher stability in Group A (T-PRF), indicating the biomaterial’s dominant effect. Age-wise analysis revealed marginally higher ISQ in younger patients (≤50 years); however, intragroup age differences were not significant (ANOVA, p > 0.05), suggesting T-PRF’s efficacy across age ranges.

Peri-implant bone density

Bone density analysis also favored T-PRF. Group A increased from 459.90 ± 23.11 HU at baseline to 685.95 ± 6.70 HU, while Group B increased from 458.10 ± 23.23 HU to 639.62 ± 9.89 HU. Intergroup differences after three months were highly significant (p < 0.001), reflecting improved bone quality with T-PRF, as observed in several studies by Aldommari et al., Ibrahim et al., and Malcangi et al. [24-26].

Mechanisms of action

The enhanced clinical outcomes associated with T-PRF can be attributed to several key mechanisms. T-PRF exhibits a dense fibrin network that provides a robust scaffold, facilitating cell migration and osteoblast activity, thereby promoting effective osseointegration and tissue regeneration [8,24]. T-PRF ensures a sustained release of growth factors, such as vascular endothelial growth factor (VEGF), platelet-derived growth factor (PDGF), and transforming growth factor beta (TGF-β), which are critical for angiogenesis, osteogenesis, and soft tissue healing [8,24,27]. The use of titanium tubes in the preparation of T-PRF minimizes the risk of contamination with silica microparticles, which have been shown to potentially cause inflammation or cytotoxicity, thereby enhancing the biocompatibility and safety of the biomaterial [28]. Collectively, these factors contribute to the superior performance of T-PRF in clinical applications.

Clinical implications

T-PRF should be considered preferentially in sinus augmentation procedures, especially in sites with low bone volume or density. Its use promotes faster and more robust osseointegration, potentially reducing healing time and the need for additional grafting procedures. The split-mouth design ensures that these effects are attributable to the biomaterial rather than interpatient variability.

Limitations

Despite the encouraging outcomes observed in this study, several limitations should be acknowledged. The follow-up period was restricted to three months, precluding assessment of long-term bone quality, implant stability, and overall success; extended observation is necessary to evaluate the durability of T-PRF-mediated effects. Bone density measurements were obtained using CBCT, which is subject to inherent variability in HU due to factors such as machine calibration, voxel size, and patient positioning, potentially introducing measurement error. Finally, while the outcome assessor was blinded to the interventions, the operator was not, which may have introduced performance bias, despite the split-mouth design controlling for interpatient variability. These limitations highlight the need for larger, long-term randomized studies with standardized protocols to confirm the reproducibility of these results.

## Conclusions

The present in vivo randomized study demonstrated that both A-PRF and T-PRF significantly enhance clinical outcomes in crestal sinus augmentation procedures when combined with bovine xenografts. However, T-PRF outperformed A-PRF in terms of implant stability (ISQ) and bone density (HU), with statistically significant improvements observed across all age and gender groups. This superior performance of T-PRF can be attributed to its denser fibrin matrix, enhanced biocompatibility, and sustained release of osteogenic growth factors.

Given the minimally invasive nature of the crestal sinus lift technique and the regenerative advantages offered by T-PRF, this study supports its use as a more effective autologous biomaterial for improving osseointegration and long-term implant success in cases with limited residual bone height. Further longitudinal studies, with larger sample sizes and histological evaluations, are recommended to validate these findings and to elucidate the underlying mechanisms at a molecular level.
